# Supplementary material for: A FAN1 point mutation associated with accelerated Huntington’s disease progression alters its PCNA-mediated assembly on DNA
Source: Nat Commun. 2025 May 14;16:4412. doi: 10.1038/s41467-025-59324-x (PMC12078616; doi:10.1038/s41467-025-59324-x)
Supplement: Supplementary file 1 — Supplementary Information [file 41467_2025_59324_MOESM1_ESM.pdf]

Supplementary information:

A FAN1 point mutation associated with accelerated Huntington's disease progression alters its PCNA-mediated assembly on DNA

Table of content

Element	Page
Supplementary table 1: Sequences of dsDNA substrates used in this study.	2
Supplementary table 2: X-ray data collection and refinement statistics.	5
Supplementary table 3: Cryo-EM data collection and refinement statistics.	6
Supplementary figure 1: Overview of protein samples used in this study.	8
Supplementary figure 2: Crystal structure of purified trFAN1 with 5′ phosphorylated p(dG)/3′(dT-dT-dT-dT) double flap DNA.	9
Supplementary figure 3: Overview of the single particle cryo-EM data processing workflow for the FAN1-DNA complex.	10
Supplementary figure 4: Overview of the single particle cryo-EM data processing workflow for the FAN1-PCNA-DNA ternary complex.	11
Supplementary figure 5: Overview of the single particle cryo-EM data processing workflow for the FAN1_R507H-PCNA-DNA ternary complex.	12
Supplementary figure 6: Overview of the single particle cryo-EM data processing workflow for the FAN1-PCNA complex bound to a (CAG)2 loop double stranded DNA.	13
Supplementary figure 7: Cryo-EM structural analysis of human FAN1, PCNA and (CAG)2 loop double stranded DNA.	14
Supplementary figure 8: FAN1 and PCNA form a ternary complex with DNA depending on the FAN1 N-terminus and the FAN1 R507 / PCNA D232 interface.	15
Supplementary figure 9: Formation of the ternary complex between FAN1, PCNA and DNA determines specificity for (CAG) extrahelical extrusion containing dsDNA.	17
Supplementary figure 10: PCNA inactivates FAN1-mediated processing of 5′pG1/3′T1 dsDNA.	19
Supplementary figure 11: PCNA activates FAN1-mediated processing of (CAG) extrahelical extrusion containing dsDNA.	21

Supplementary Tables

Supplementary table 1: Sequences of dsDNA substrates used in this study.

dsDNA substrate	sequence	modification
**cryo-EM DNA substrate**	CCC GTC CAG GTC TCG TCC GCG CCA CTC GTG TCC AGC GTC G	
	TGC GGA CGA GAC CTG GAC GGG	5′-phosphate
	CGA CGC TGG ACA CGA GTG GCT TTT TTT T	
**crystallography DNA**	AAC ACG CCT AGA CTC CTC A	--
	TTT GAG GAG TCT TTT T	--
	GAG GCG TG	5′-phosphate
**MST DNA**	CGA CGC TGG ACA CGA GTG GCT	
	CCC GTC CAG GTC TCG TCC GCG CCA CTC GTG TCC AGC GTC G	
	GCG GAC GAG ACC TGG ACG GG	5′-phosphate
**61 bp homoduplex**	TAG CAA GCT GCA GCC AGC CGC CTA GAA ATT CGG CTT TC	
	GAATTGGGTACCGCTGAATTGCACCGAGCTGATCCTCGA TGATCCTAAGCTAAGCTTCAG	Non/5′-biotin/3′-Cy5
**61 bp (CAG)1 extrahelical extrusion**	CTGAAGCTTAGCTTAGGATCATCGAGGATCCAGAGCTCG GTGCAATTCAGCGGTACCCAATTC	
	GAATTGGGTACCGCTGAATTGCACCGAGCTGATCCTCGA TGATCCTAAGCTAAGCTTCAG	Non/5′-biotin/3′-Cy5
**61 bp (CAG)2 extrahelical extrusion**	CTGAAGCTTAGCTTAGGATCATCGAGGATCCAGCAGAGC TCGGTGCAATTCAGCGGTACCCAATTC	
	GAATTGGGTACCGCTGAATTGCACCGAGCTGATCCTCGA TGATCCTAAGCTAAGCTTCAG	Non/5′-biotin/3′-Cy5
**61 bp (CAG)3 extrahelical extrusion**	CTGAAGCTTAGCTTAGGATCATCGAGGATCCAGCAGCAG AGCTCGGTGCAATTCAGCGGTACCCAATTC	
	GAATTGGGTACCGCTGAATTGCACCGAGCTGATCCTCGA TGATCCTAAGCTAAGCTTCAG	Non/5′-biotin/3′-Cy5
**61 bp (CAG)4 extrahelical extrusion**	CTG AAG CTT AGC TTA GGA TCA TCG AGG ATC CAG CAG CAG CAG AGC TCG GTG CAA TTC AGC GGT ACC CAA TTC	
	GAATTGGGTACCGCTGAATTGCACCGAGCTGATCCTCGA TGATCCTAAGCTAAGCTTCAG	Non/5′-biotin/3′-Cy5

**113 bp homoduplex**	CCG CTT TCT TCC CTT CCT TTG TGC ACA CGT TCC GAG ATA TCC TAG CAA GTG ATC GTC TAT GTA GCT CAA GAG TTC GAC TTT CCC CGC TAA GCT CTA CAT CCG AGG CTC GCC GA	
	TCG GCG AGC CTC GGA TGT AGA GCT TAG CGG GGA AAG TCG AAC TCT TGA GCT ACA TAG TAC GAT CAC TTG CTA GGA TAT CTC GGA ACG TGT GCA CAA AGG AAG GGA AGA AAG CGG	5′-biotin
**113 bp (CAG)1 extrahelical extrusion**	CCG CTT TCT TCC CTT CCT TTG TGC ACA CGT TCC GAG ATA TCC TAG CAA GTG ATC GTC AGC TAT GTA GCT CAA GAG TTC GAC TTT CCC CGC TAA GCT CTA CAT CCG AGG CTC GCC GA	
	TCG GCG AGC CTC GGA TGT AGA GCT TAG CGG GGA AAG TCG AAC TCT TGA GCT ACA TAG TAC GAT CAC TTG CTA GGA TAT CTC GGA ACG TGT GCA CAA AGG AAG GGA AGA AAG CGG	5′-biotin
**113 bp (CAG)2 extrahelical extrusion**	CCG CTT TCT TCC CTT CCT TTG TGC ACA CGT TCC GAG ATA TCC TAG CAA GTG ATC GTC AGC AGC TAT GTA GCT CAA GAG TTC GAC TTT CCC CGC TAA GCT CTA CAT CCG AGG CTC GCC GA	
	TCG GCG AGC CTC GGA TGT AGA GCT TAG CGG GGA AAG TCG AAC TCT TGA GCT ACA TAG TAC GAT CAC TTG CTA GGA TAT CTC GGA ACG TGT GCA CAA AGG AAG GGA AGA AAG CGG	5′-biotin
**113 bp (CAG)3 extrahelical extrusion**	CCG CTT TCT TCC CTT CCT TTG TGC ACA CGT TCC GAG ATA TCC TAG CAA GTG ATC GTC AGC AGC AGC TAT GTA GCT CAA GAG TTC GAC TTT CCC CGC TAA GCT CTA CAT CCG AGG CTC GCC GA	
	TCG GCG AGC CTC GGA TGT AGA GCT TAG CGG GGA AAG TCG AAC TCT TGA GCT ACA TAG TAC GAT CAC TTG CTA GGA TAT CTC GGA ACG TGT GCA CAA AGG AAG GGA AGA AAG CGG	5′-biotin
**113 bp (CAG)4 extrahelical extrusion**	CCG CTT TCT TCC CTT CCT TTG TGC ACA CGT TCC GAG ATA TCC TAG CAA GTG ATC GTC AGC AGC AGC AGC TAT GTA GCT CAA GAG TTC GAC TTT CCC CGC TAA GCT CTA CAT CCG AGG CTC GCC GA	
	TCG GCG AGC CTC GGA TGT AGA GCT TAG CGG GGA AAG TCG AAC TCT TGA GCT ACA TAG TAC GAT CAC TTG	5′-biotin

	CTA GGA TAT CTC GGA ACG TGT GCA CAA AGG AAG GGA<br><br>AGA AAG CGG	

Supplementary table 2: X-ray data collection and refinement statistics. Values in parenthesis refer to the highest resolution shell

**Crystal structure of FAN1 nuclease bound to 5′-phosphorylated p(dG)/3′(dT-dT-dT-dT) double flap DNA**	
PDB ID	8S5A
Data processing statistics	
X-ray source	CMCF-ID
Wavelength [Å]	0.9537
Detector	EIGER
Temperature [K]	100
Spacegroup	$P2_12_12_1$
Cell:	
a; b; c [Å]	91.63; 100.55; 112.88
a; b; g [°]	90.0; 90.0; 90.0
Resolution [Å]	43.357 – 2.645 (2.691-2.645)
Unique reflections	31091 (1538)
Multiplicity	11.2 (11.8)
Completeness	99.9 (99.9)
Mn(I)/sigma	16.6 (1.4)
Rsym[%]	6.7 (124.2)
Rmeas [%]	6.7 (129.7)
Refinement statistics	
Resolution (Å)	43.39 – 2.65
Number of reflections	30267
R work /free (%)	23.4 / 27.3
Model composition	
Protein atoms	4785
Nucleic acid atoms	816
Heterogen atoms	3
Solvent atoms	16
R.m.s. deviations	
Bond lengths (Å)	0.003
Bond angles (°)	1.249
Validation	
MolProbity score	1.15
Clashscore	1.44
Poor rotamers (%)	0.8
Ramachandran plot	
Favored (%)	96.56
Allowed (%)	3.44
Disallowed (%)	0.00

Supplementary table 3: Cryo-EM data collection and refinement statistics.

**PDB ID**	**9EOA**	**9EO1**	**9GY0**
Sample conditions			
Sample concentration		3 μM of each component	
Grid type		R1.2/1.3 Au/Cu 300 mesh, Quantifoil	
Cryo-EM data collection			
Microscope		Glacios transmission electron microscope (Thermo Fisher)	
Voltage (kV)		200	
Spherical aberration Cs (mm)		2.7	
Condenser C2 aperture size (μm)		100	
Objective aperture size (μm)		100	
Camera		Falcon 4 electron detector	
Pixel size (Å)		0.9142	
Total dose (electron*$Å^{-2}$)	51,55	49,95	59,95
Images per hole	1	1	2
Defocus range (μm)	0.8 – 2.2	0.6 – 1.8	0.8 - 2.2
# micrographs collected	6,289	5,978	4664
% micrographs used	80.8	82.4	89
Cryo-EM data processing			
Software		cryoSPARC v4.1.2	
Picked particles	~2.3 million	~1.1 million	~3 million
Particles after 2D classification	~1.3 million	679,040	831,038
Symmetry	C1	C1	C1
Particles after 3D sorting	700,432	379,066	487,606
Resolution (FSC 0.143, Å)	3.27	3.2	3.42
Model building and refinement			
Software for building		Coot 0.9.4.7 EL	
Residues build		31-556	
Software for refinement		PHENIX 1.20.1 - 4487	
Composition (#)			

Chains	4	7	7
Atoms	5957 (Hydrogens: 0)	11807 (Hydrogens: 0)	11866 (Hydrogens: 0)
Residues	Protein: 576	Protein: 1337	Protein: 1340
	Nucleotide: 63	Nucleotide: 63	Nucleotide: 65
Bonds (RMSD)			
Length (Å) (# > 4σ)	0.006	0.006	0.004 (3)
Angles (°) (# > 4σ)	0.859 (7)	0.856 (10)	0.632 (5)
Validation			
MolProbity score	2.34	2.40	2.19
Clashscore	17.79	18.32	14.55
Rotamer outliers (%)	3.16	3.84	2.21
Ramachandran plot			
Favored (%)	96.65	96.81	96.21
Allowed (%)	3.35	3.19	3.79
Outliers (%)	0.00	0.00	0.00

Supplementary figures

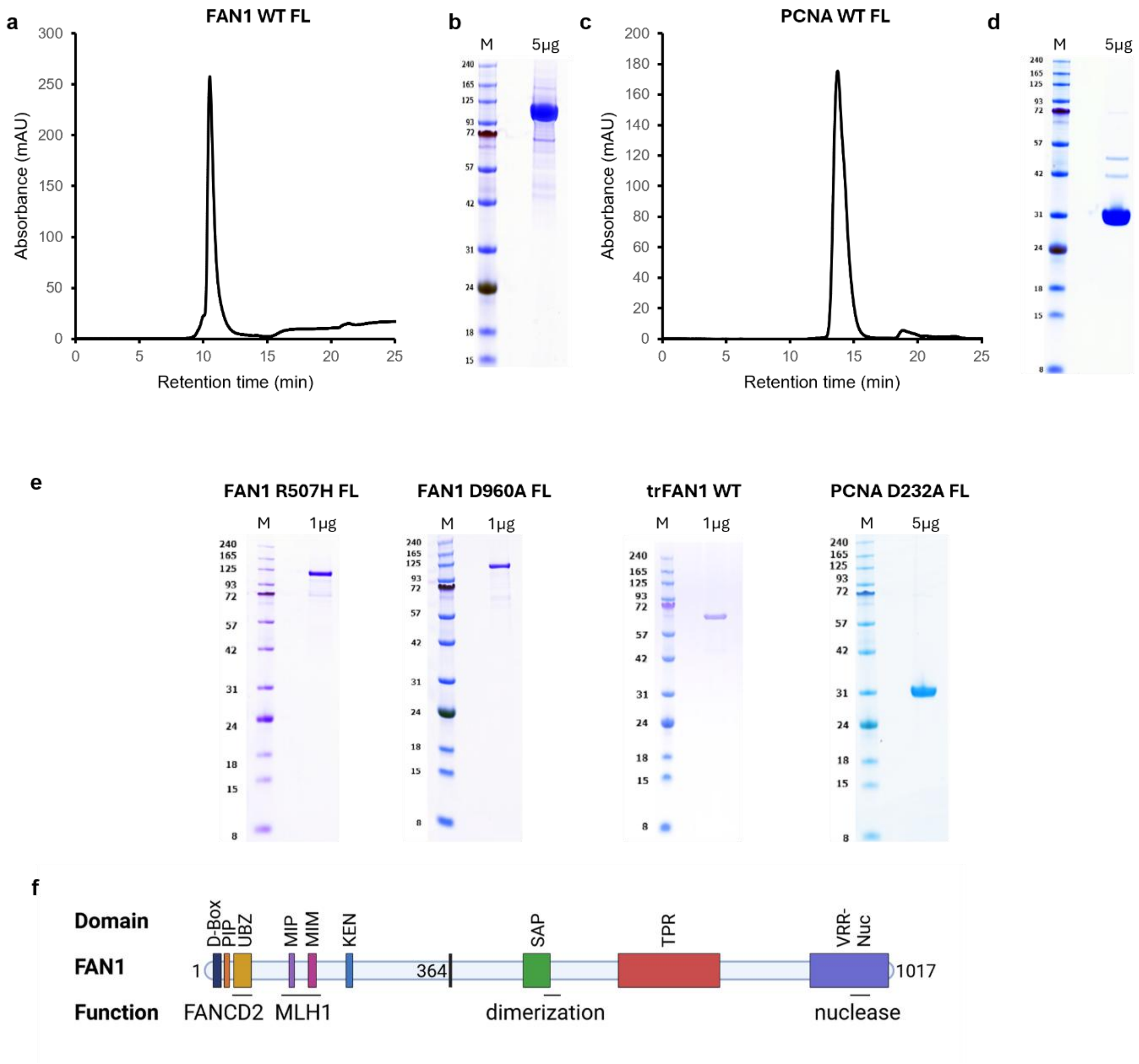

Supplementary figure 1: Overview of protein samples used in this study. Representative final SEC profiles and 5 μg gels are shown for (A, B) FAN1 and (C, D) PCNA after final rebuffering before complexation for cryo-EM studies. (E) 5 μg gels of FAN1 and PCNA variants used in this study in biochemical and biophysical assays. (F) Domain architecture of FAN1 annotated with corresponding function.

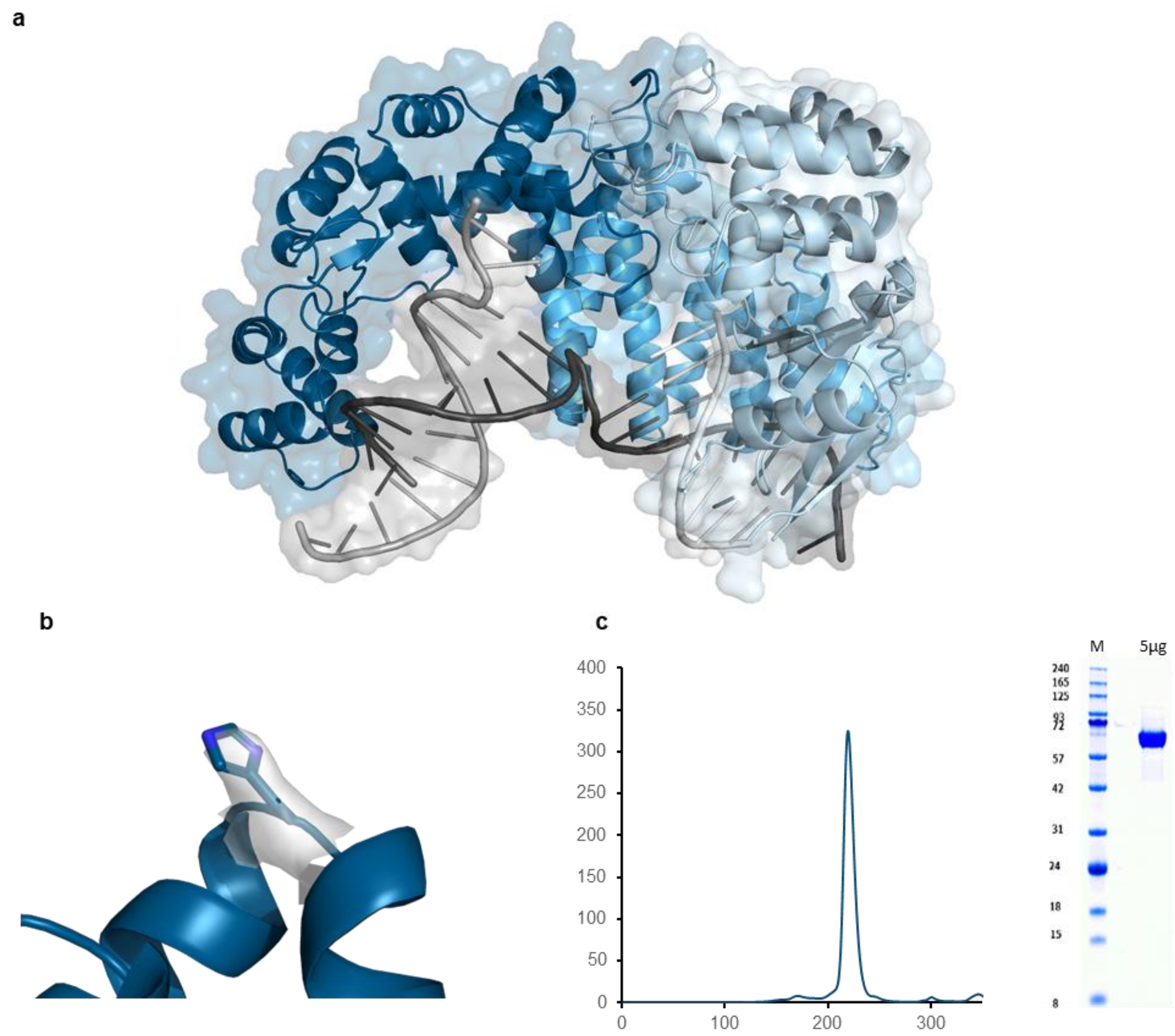

Supplementary figure 2: Crystal structure of purified trFAN1 (364-1017, R507H, K794A, Δ510-518) with 5′ phosphorylated p(dG)/3′(dT-dT-dT-dT) double flap DNA. (A) The overall structure of Fanconi-associated nuclease 1 (FAN1) in complex with double flap DNA consists of a SAP domain (SAP, residues 371 – 594, medium blue), a middle tetratricopeptide repeat domain (TPR domain, residues 595- 772, dark blue) and a C-terminal viral replication and repair nuclease domain (VRR_nuc domain, residues 773 – 1010, light blue) and folds into the shape of a short-handle scoop. (B) *2FoFc* electron density contoured at 1σ for H507. (C) Final SEC profile and 5 µg SDS-PAGE of protein used for crystallographic studies.

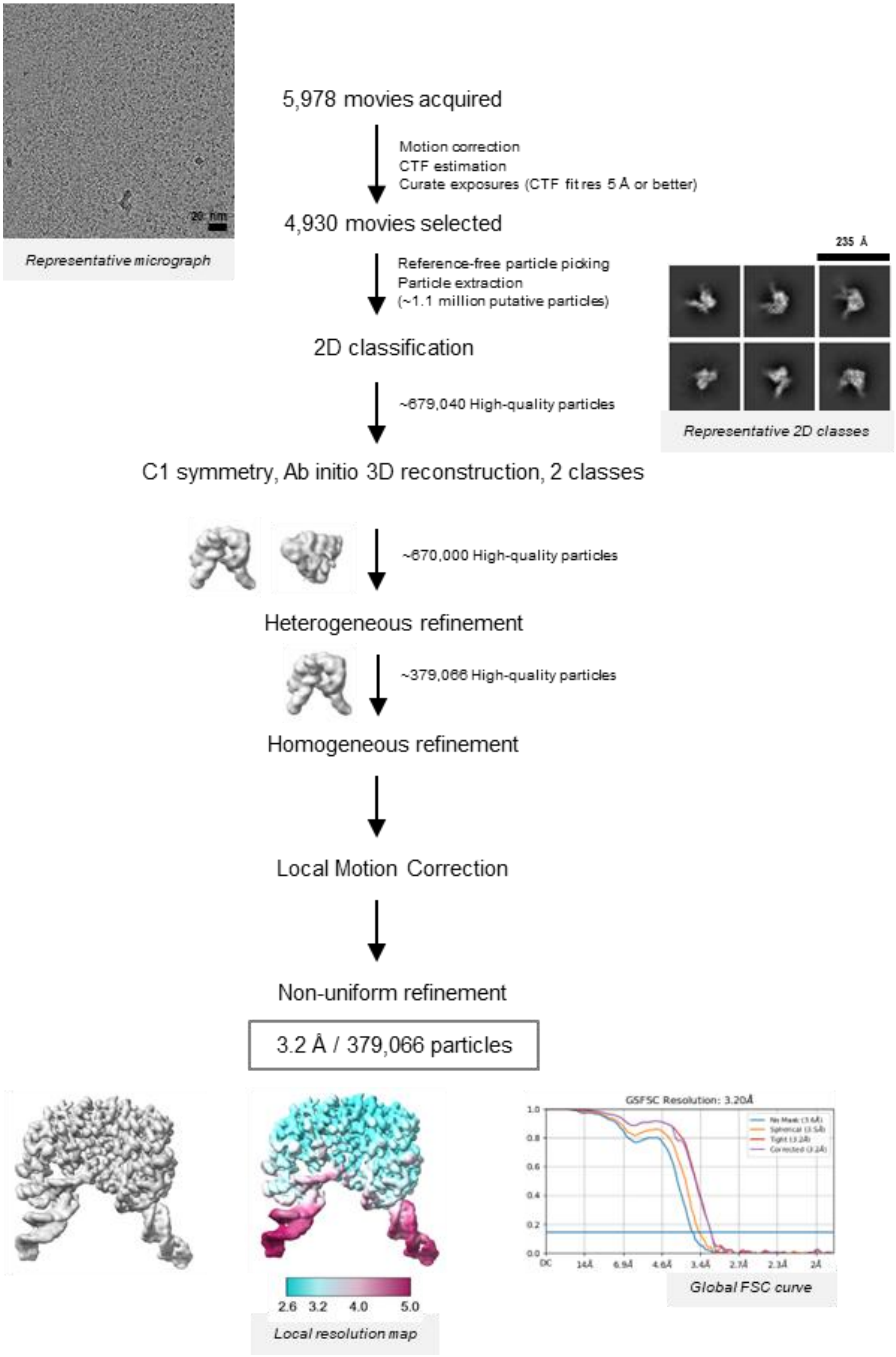

Supplementary figure 3: Overview of the single particle cryo-EM data processing workflow for the FAN1-DNA complex. Also see Methods.

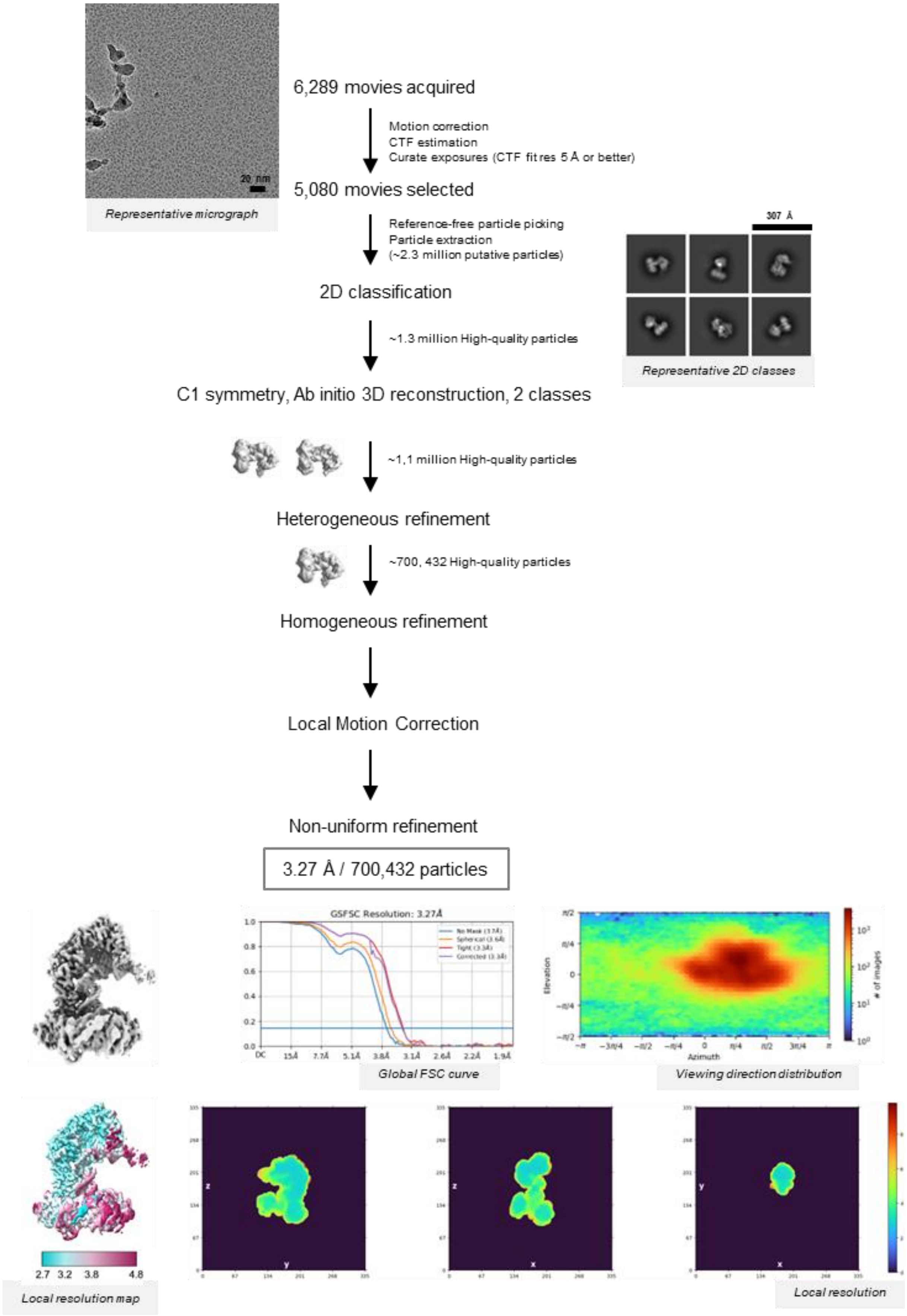

Supplementary figure 4: Overview of the single particle cryo-EM data processing workflow for the FAN1-PCNA-DNA ternary complex. Also see Methods.

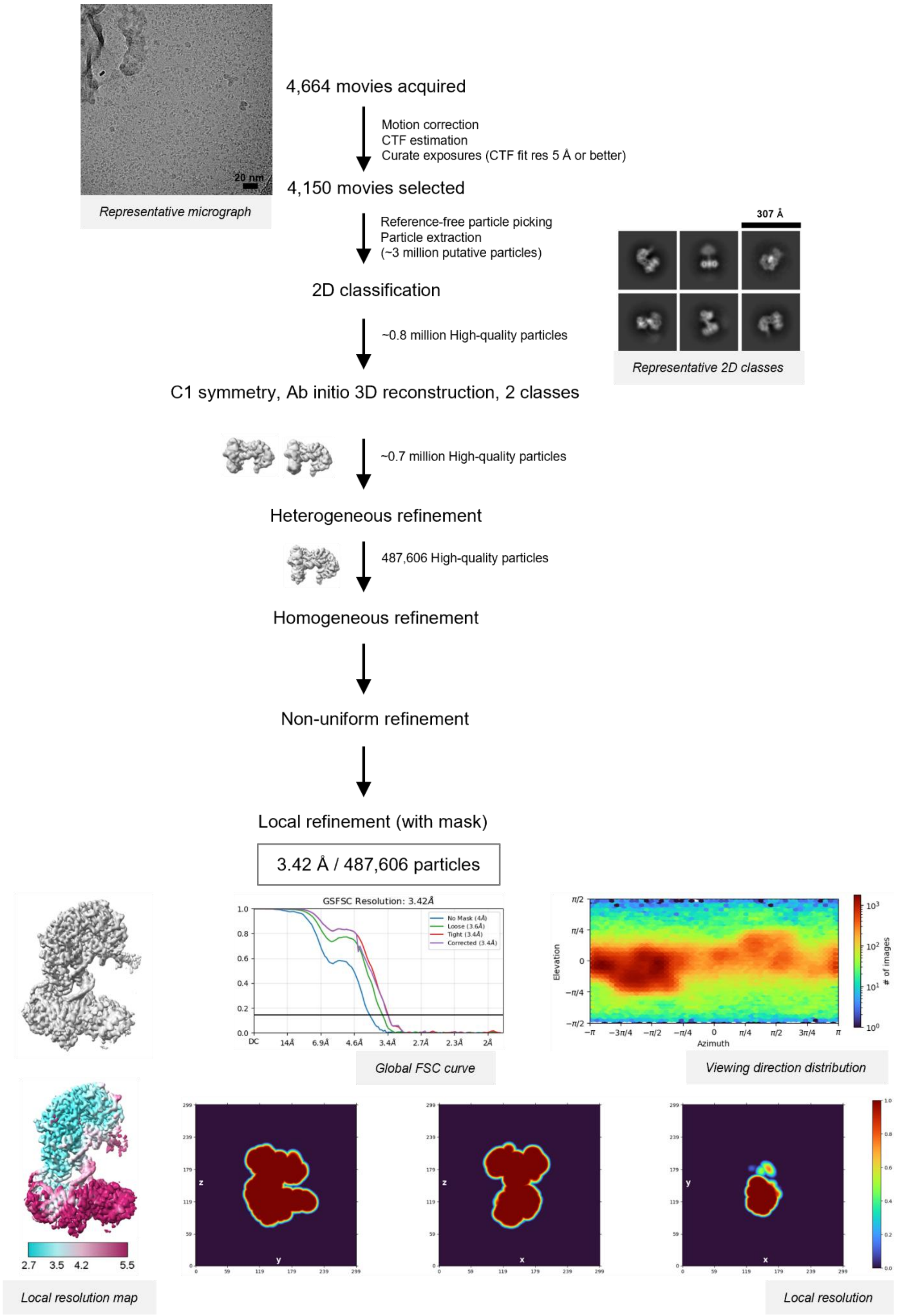

Supplementary figure 5: Overview of the single particle cryo-EM data processing workflow for the FAN1_R507H-PCNA-DNA ternary complex. Also see Methods.

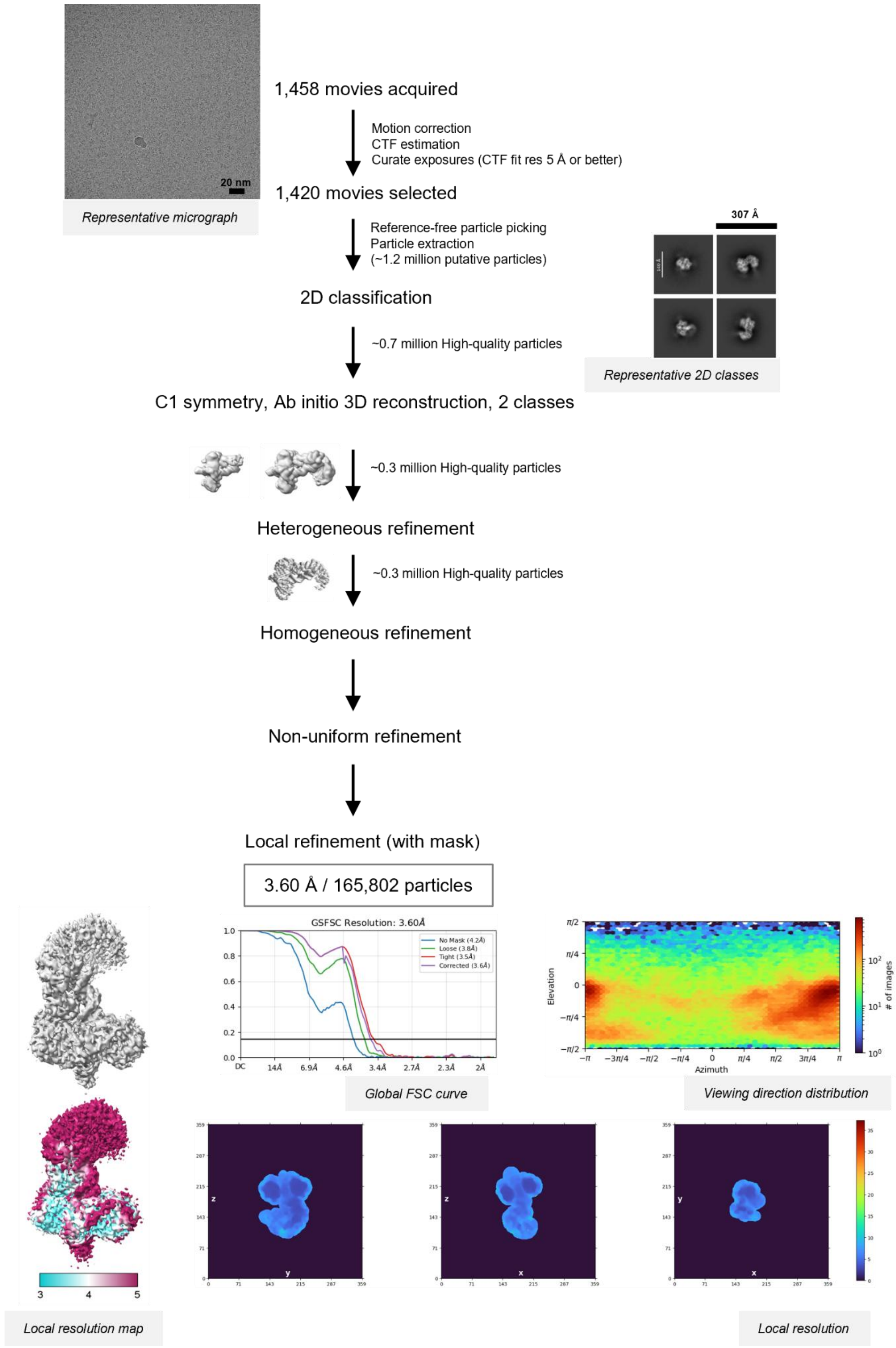

Supplementary figure 6: Overview of the single particle cryo-EM data processing workflow for the FAN1-PCNA complex bound to a (CAG)2 loop double stranded DNA. Also see Methods.

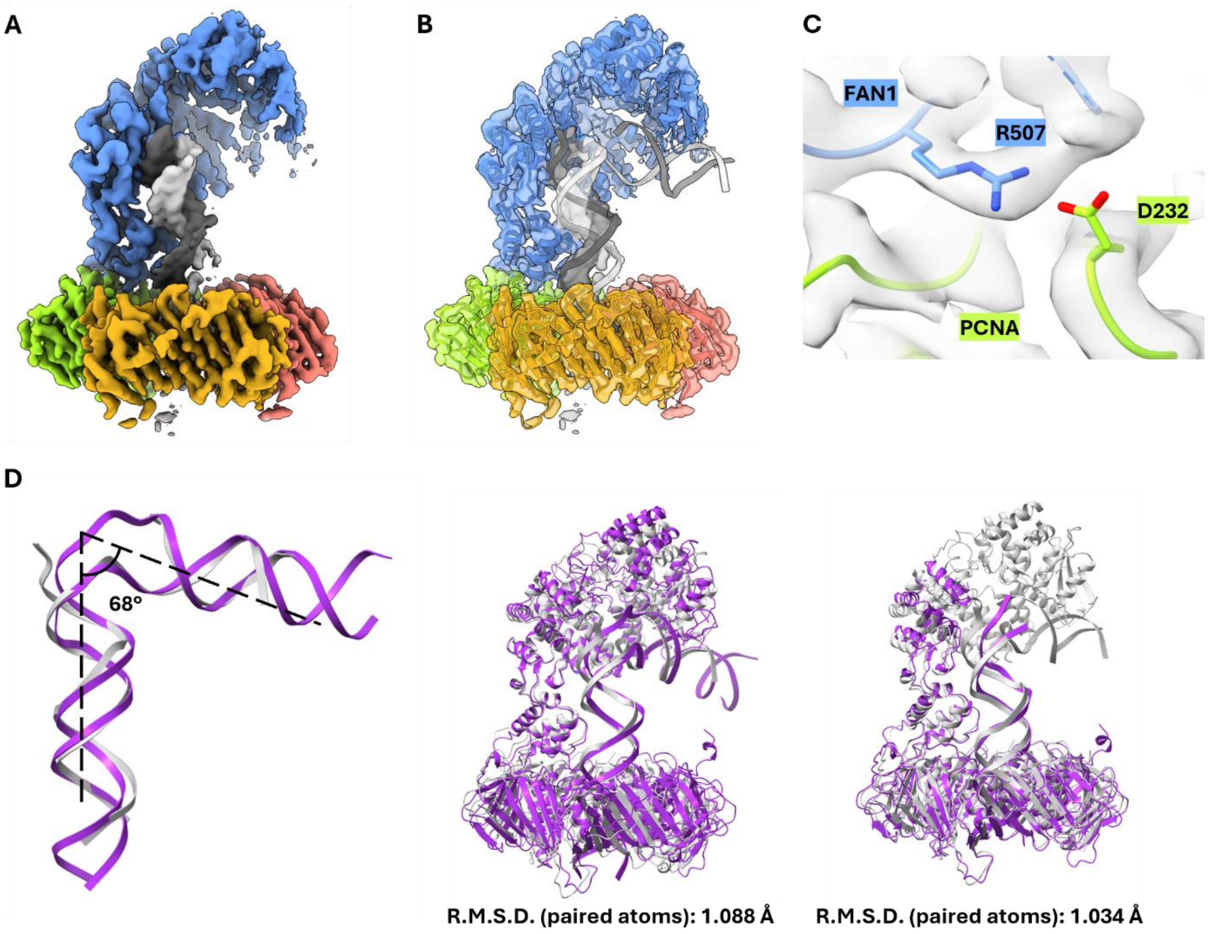

Supplementary figure 7: Cryo-EM structural analysis of human FAN1, PCNA and (CAG)2 loop double stranded DNA. (A) Cryo-EM density map of FAN1-PCNA-(CAG)2 loop DNA at 3.6 Å resolution. FAN1 is depicted in blue, PCNA in yellow/red/green, and DNA in gray/white. (B) Rigid-body fit of the cryo-EM density map of FAN1-PCNA-(CAG)2 loop DNA with the previously resolved ternary complex of FAN1-PCNA-5′ flap DNA (PDB: 9EO1). The cryo-EM density map is shown in surface as transparent density overlayed with a cartoon representation of the model. (C) Close-up view of FAN1-PCNA interface of the rigid-body fit model of 9EO1 in FAN1-PCNA-(CAG)2 loop DNA density map. The density for R507 of FAN1 is visible and extends towards D232 of PCNA. (D) Overall structural comparison between FAN1-PCNA-5'flap DNA (gray) (9EO1) and FAN1-PCNA-(CAG)2 loop DNA (dark orchid) complexes (left: 9CG4, right: 9CL7)[30] reveals no conformational changes at the FAN1-PCNA interface or in the DNA trajectory. Root-mean-square deviation (RMSD) values for the paired atoms confirm the structural conservation across different substrates.

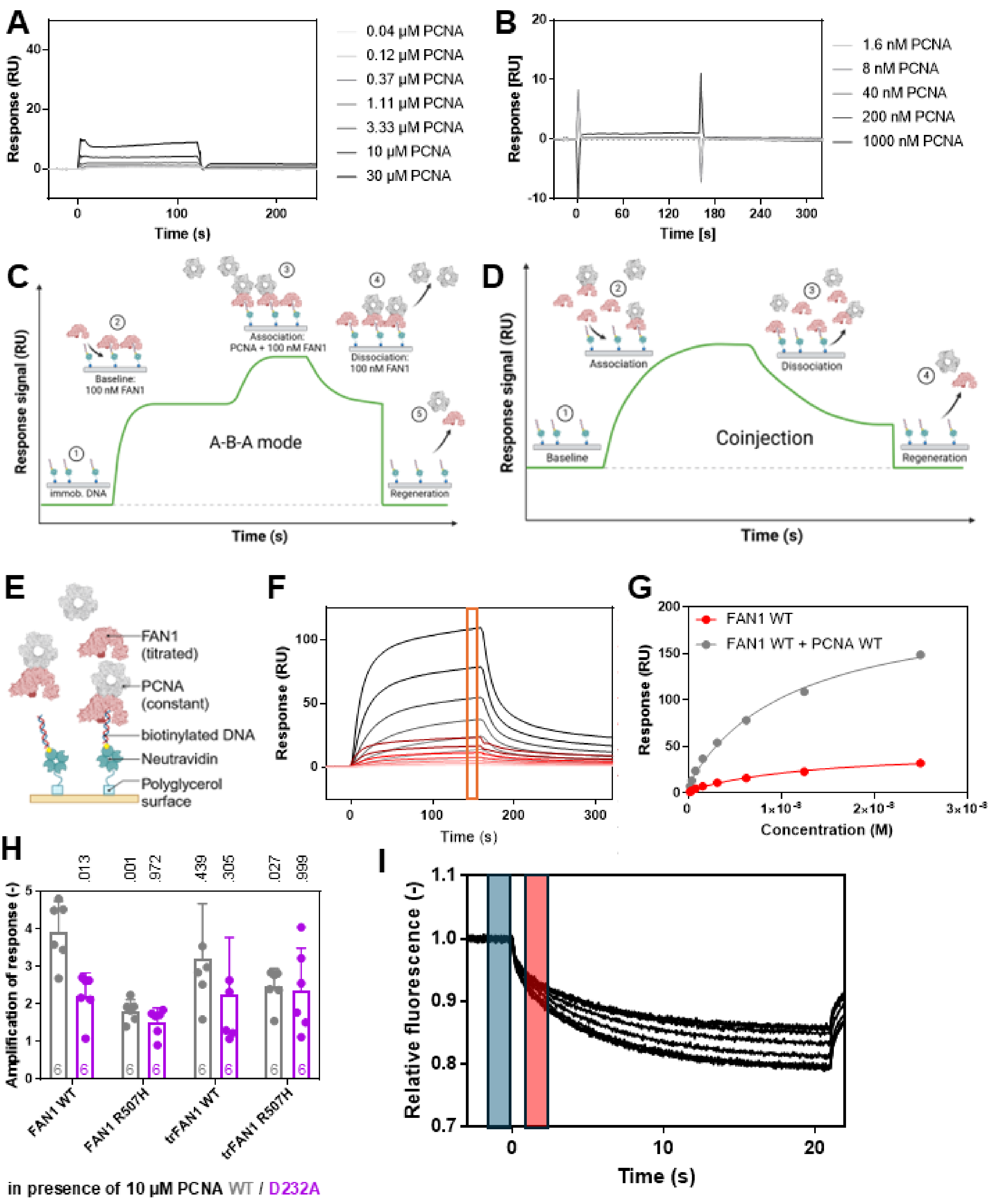

Supplementary figure 8: FAN1 and PCNA form a ternary complex with DNA depending on the FAN1 N-terminus and the FAN1 R507 / PCNA D232 interface. A) Exemplary sensorgrams of increasing PCNA concentrations binding to FAN1. Fast binding kinetics and low responses indicate affinity in the high micromolar range. B) Exemplary sensorgrams of increasing PCNA concentrations binding to biotinylated 113 bp homoduplex dsDNA. Fast binding kinetics and low responses indicate

affinity in the high micromolar range. C, D) Schemes of the applied SPR assay setups to study ternary PCNA – FAN1 – DNA complex formation. For both setups, neutravidin is covalently immobilized onto a carboxylated polyglycerol (CMPG) sensor chip surface. Subsequently, biotinylated DNA is immobilized. C) For PCNA titrations, each measurement cycle starts with (1) a baseline, followed by (2) an injection of FAN1 in running buffer to allow formation of a FAN1 – DNA complex. (3) In the association phase, different concentrations of PCNA are injected in presence of FAN1 to determine the on-rate (k_{on}). Interactions with a fast on-rate will reach equilibrium during the association phase. The (4) dissociation phase is recorded in presence of FAN1 to determine the PCNA off-rate (k_{off}). Finally, the surface is (5) regenerated with SDS. D) For FAN1 titrations, a regular sensorgram is recorded consisting of (1) baseline, (2) association, (3) dissociation and (4) regeneration phase with FAN1 being titrated in presence and absence of PCNA. Due to mixed kinetics originating in association and dissociation of FAN1 or the FAN1 – PCNA complex, no kinetic analysis can be performed with this setup due to deviation from a 1:1 binding model and hence, only steady state responses after reaching equilibrium towards the end of the association phase were analyzed. E) Scheme of the SPR assay in which FAN1 was titrated in the presence of PCNA using coinjections (**Supplementary figure 8D, F-H**). F) Exemplary sensorgrams of increasing FAN1 WT concentrations binding to 113 bp homoduplex DNA in presence (grey) and absence (red) of 10 µM PCNA WT. Responses for steady state affinity determination were read out 5 s before injection end (orange box). G) Exemplary steady state affinity of FAN1 WT binding to 113 bp homoduplex DNA in presence (grey) and absence (red) of PCNA. Data is fitted with a one-site binding model (lines). H) Amplification of responses were determined from steady state affinity fits by dividing the maximal response in presence of PCNA WT (grey) or PCNA D232A (purple) by the maximal response in absence of PCNA for (tr)FAN1 WT and R507H. I) Exemplary MST traces of a titration of PCNA WT to Cy5-labeled 61 bp homoduplex DNA in presence of FAN1 WT. Data was analyzed 1.5 s after turning on the infrared laser (red box) and compared to the baseline before heating the sample (blue box). The MST traces show no sign of aggregation indicating a high quality of the applied protein samples. Schemes in figure created in BioRender. Aret, J. (2024) BioRender.com/m48p024

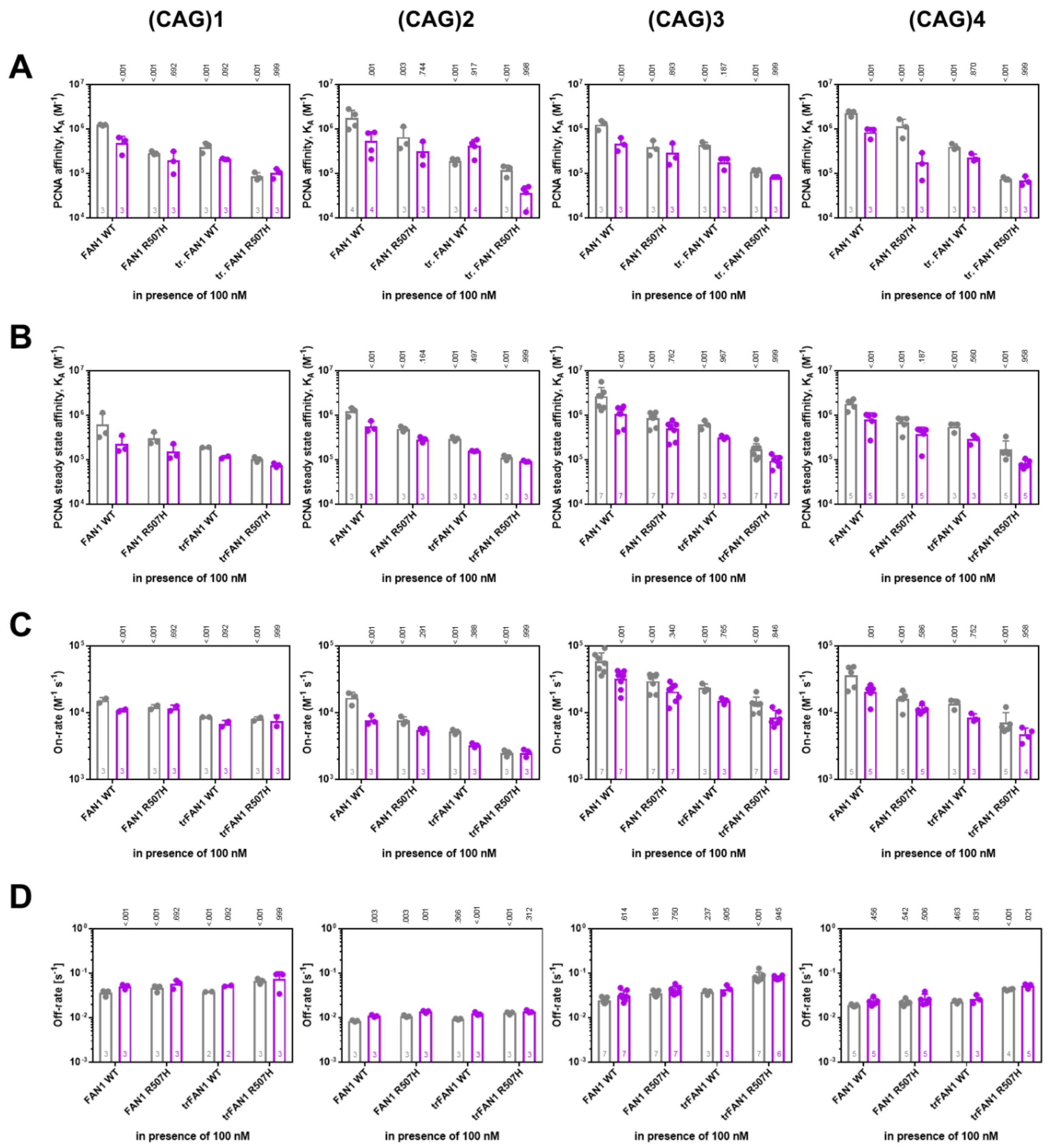

Supplementary figure 9: Formation of the ternary complex between FAN1, PCNA and DNA determines specificity for (CAG) extrahelical extrusion containing dsDNA. A) MST-derived affinities for PCNA WT (grey) and D232A (purple) binding in presence of 100 nM (tr)FAN1 WT and R507H to Cy5-labeled 61 bp (CAG)1, (CAG)2, (CAG)3 or (CAG)4 extrahelical extrusion containing dsDNA (from left to right). B) SPR-derived steady state affinities for PCNA WT (grey) and D232A (purple) binding in presence of 100 nM (tr)FAN1 WT and R507H to immobilized, biotinylated 113 bp (CAG)1, (CAG)2, (CAG)3 or (CAG)4 extrahelical extrusion containing dsDNA (from left to right). C, D) SPR-derived C) on-rates and D) off-rates for PCNA WT (grey) and D232A (purple) binding in presence of 100

nM (tr)FAN1 WT and R507H to immobilized, biotinylated 113 bp (CAG)1, (CAG)2, (CAG)3 or (CAG)4 extrahelical extrusion containing dsDNA (from left to right). Bar charts represent the mean and error bars the standard deviation of *n* independent measurements. P values are indicated above the bars comparing values to measurements in presence of FAN1 WT for PCNA WT titrations using a two-way ANOVA with Dunnett's post hoc test and measurements with PCNA D232A are compared to PCNA WT measurements using a two-way ANOVA with Sidak's post hoc test.

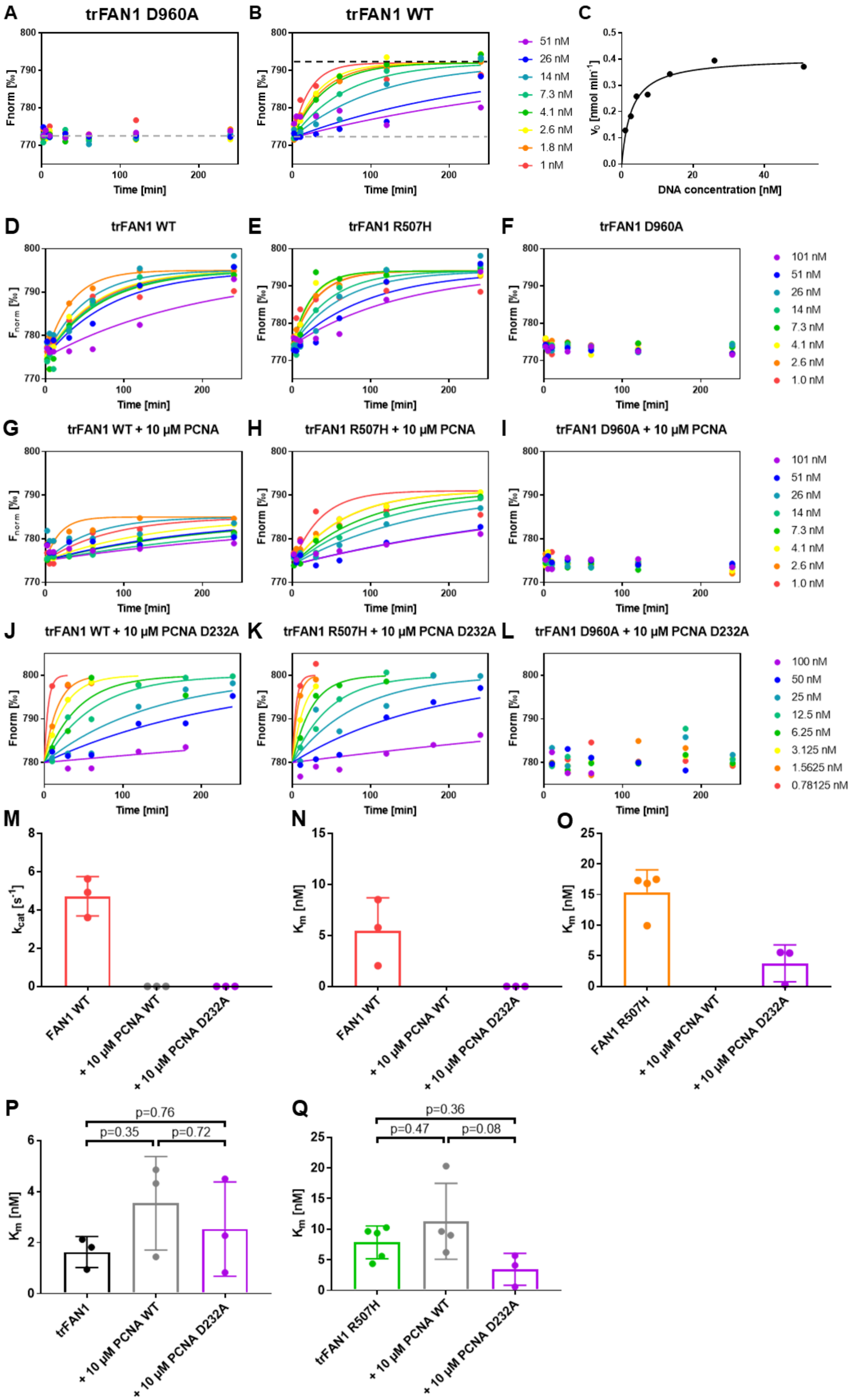
A
trFAN1 D960A
B
trFAN1 WT
C
Fnorm [‰]
Time [min]
51 nM
26 nM
14 nM
7.3 nM
4.1 nM
2.6 nM
1.8 nM
1 nM
v0 [nmol min-1]
DNA concentration [nM]
D
trFAN1 WT
E
trFAN1 R507H
F
trFAN1 D960A
101 nM
51 nM
26 nM
14 nM
7.3 nM
4.1 nM
2.6 nM
1.0 nM
G
trFAN1 WT + 10 µM PCNA
H
trFAN1 R507H + 10 µM PCNA
I
trFAN1 D960A + 10 µM PCNA
J
trFAN1 WT + 10 µM PCNA D232A
K
trFAN1 R507H + 10 µM PCNA D232A
L
trFAN1 D960A + 10 µM PCNA D232A
100 nM
50 nM
25 nM
12.5 nM
6.25 nM
3.125 nM
1.5625 nM
0.78125 nM
M
N
O
kcat [s-1]
Km [nM]
FAN1 WT
+ 10 µM PCNA WT
+ 10 µM PCNA D232A
FAN1 R507H
P
Q
p=0.76
p=0.35
p=0.72
p=0.36
p=0.47
p=0.08
trFAN1
trFAN1 R507H

Supplementary figure 10: PCNA inactivates FAN1-mediated processing of 5′pG1/3′T1 dsDNA. A-C) For the data analysis A) a baseline corresponding to the signal of unreacted DNA was determined from the average signal of a time course experiment with a catalytically dead FAN1 D960A mutant (grey line) and the B) plateau corresponding to the signal of reacted DNA (black line) was determined from time course experiment with an active FAN1 using the values of low DNA concentrations after 4 h reaction time. The time course data was fitted to a one-phase association curve setting the plateau and baseline to the previously determined values for reacted and unreacted DNA, respectively. The difference between plateau and baseline was typically ΔF_{norm} = 20‰. The obtained reaction rates K were multiplied with the DNA concentration in the sample to determine the initial reaction rate v_0, that was C) plotted against the DNA concentration and fitted to a Michaelis Menten model to determine K_m and the limiting rate v_{max}. D-L) Exemplary activity data of D, G, J) trFAN1 WT, E, H, K) trFAN1 R507H and F, I, L) trFAN1 D960A in D-F) absence and presence of G-I) 10 µM PCNA WT or J-L) 10 µM PCNA D232A. M) Catalytic efficiency (k_{cat}) of FAN1 WT (red) in presence and absence of 10 µM PCNA WT (grey) or PCNA D232A (purple; n=3). Note that no activity was detected in the presence of PCNA. N-Q) K_m values for 5′pG1/3′T1 dsDNA of N) FAN1 WT (red) O) FAN1 R507H (orange), P) trFAN1 WT (black), and Q) trFAN1 R507H (green) in presence and absence of 10 µM PCNA WT (grey) or PCNA D232A (purple; n≥3; p-values were calculated using an unpaired, two-tailed t-test with Welch’s correction).

:

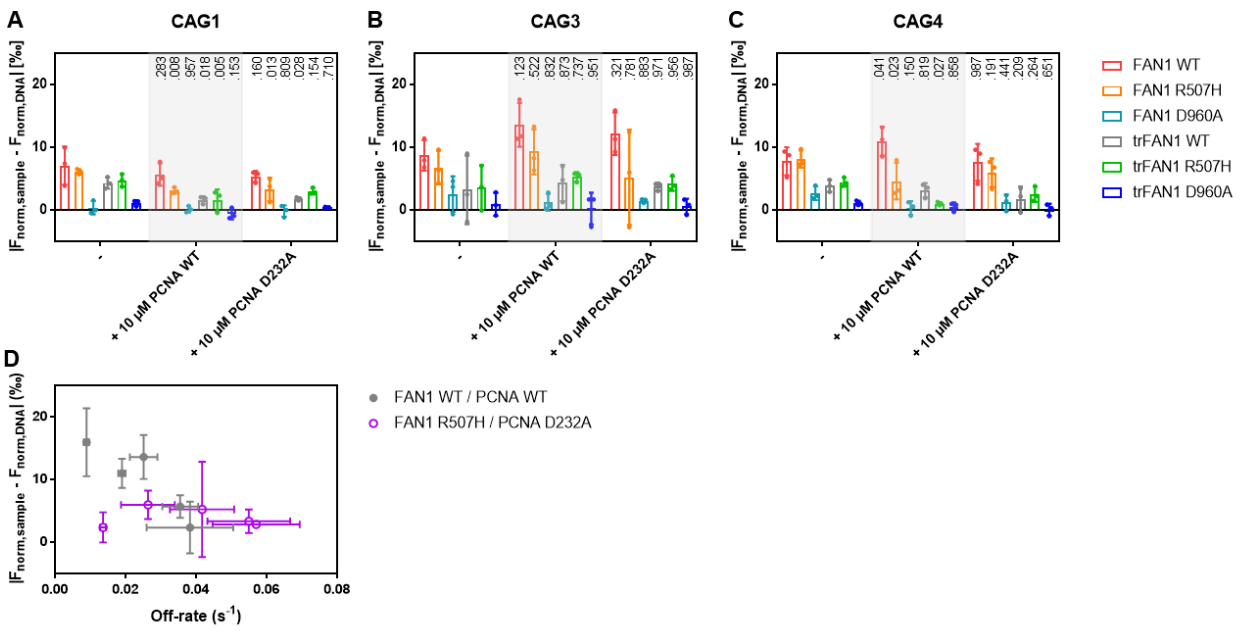

Supplementary figure 11: PCNA activates FAN1-mediated processing of (CAG) extrahelical extrusion containing dsDNA. A-C) Changes in MST signal in presence of 50 nM FAN1 WT (red), FAN1 R507H (orange), FAN1 D960A (light blue), trFAN1 WT (grey), trFAN1 R507H (green) and trFAN1 D960A (dark blue) compared to free DNA ($|F_{norm,sample} - F_{norm,DNA}|$) after 30 min incubation at 37°C for A) 1 nM Cy5-labeled 61 bp (CAG)1 extrahelical extrusion containing DNA, B) 1 nM Cy5-labeled 61 bp (CAG)3 extrahelical extrusion containing DNA and C) 1 nM Cy5-labeled 61 bp (CAG)4 extrahelical extrusion containing DNA in presence and absence of 10 µM PCNA WT or D232A. P values are indicated above the bars comparing values to in presence of PCNA with measurements in absence of PCNA using a two-way ANOVA with Dunnett's post hoc test. Bar charts represent the mean and all error bars the standard deviation of *n=3* independent measurements. D) Correlation between MST-derived activity in presence of FAN1 WT and PCNA WT (grey, **Figure 5 H**) or FAN1 R507H and PCNA D232A (purple) with SPR-derived off-rates for PCNA WT binding in presence of FAN1 WT (grey, see **Figures 3 and 4**) or PCNA D232A binding in presence of FAN1 R507H (purple) for homoduplex or (CAG)1, (CAG)2, (CAG)3 or (CAG)4 extrahelical extrusion containing dsDNA substrates.
